# Orientation-Selective and Frequency-Correlated Light-Induced Pulsed Dipolar Spectroscopy

**DOI:** 10.1021/acs.jpclett.1c00595

**Published:** 2021-04-15

**Authors:** Alice M. Bowen, Arnau Bertran, Kevin B. Henbest, Marina Gobbo, Christiane R. Timmel, Marilena Di Valentin

**Affiliations:** † Department of Chemistry, Photon Science Institute and The National EPR Research Facility, The University of Manchester, Oxford Road, Manchester M13 9PL, United Kingdom; ‡ Centre for Advanced Electron Spin Resonance and Inorganic Chemistry Laboratory, Department of Chemistry, University of Oxford, South Parks Road, Oxford OX1 3QR, United Kingdom; § Department of Chemical Sciences, University of Padova, Via Marzolo 1, 35131 Padova, Italy

## Abstract

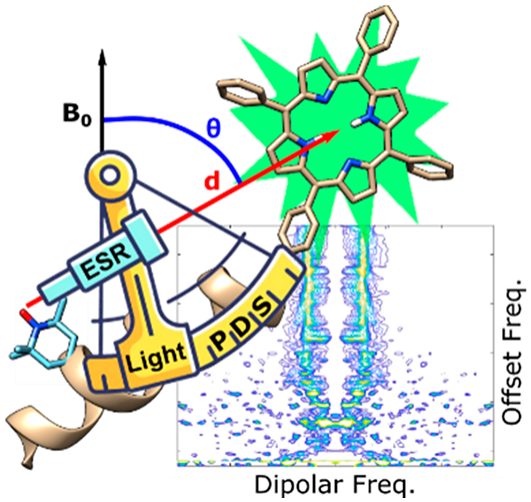

We explore the potential of orientation-resolved pulsed dipolar spectroscopy (PDS) in light-induced versions of the experiment. The use of triplets as spin-active moieties for PDS offers an attractive tool for studying biochemical systems containing optically active cofactors. Cofactors are often rigidly bound within the protein structure, providing an accurate positional marker. The rigidity leads to orientation selection effects in PDS, which can be analyzed to give both distance and mutual orientation information. Herein we present a comprehensive analysis of the orientation selection of a full set of light-induced PDS experiments. We exploit the complementary information provided by the different light-induced techniques to yield atomic-level structural information. For the first time, we measure a 2D frequency-correlated laser-induced magnetic dipolar spectrum, and we are able to monitor the complete orientation dependence of the system in a single experiment. Alternatively, the summed spectrum enables an orientation-independent analysis to determine the distance distribution.

Electron spin resonance (ESR) pulsed dipolar spectroscopy (PDS) is an invaluable biophysical technique for studying complex biological assemblies.^1−5^ The dipolar interaction between two moieties with nonzero electronic spin is measured as an oscillating time trace. Analysis and simulation of these time traces can reveal the relative distance and, for rigid systems, orientation distributions of the two moieties,^6−10^ providing information about the structure and conformation of the (bio)molecule(s) to which these moieties are attached. Typical ESR PDS techniques, such as double electron–electron resonance (DEER),^11^ also known as pulsed electron double resonance (PELDOR),^12^ are used to study systems containing stable centers with nonzero electronic spin. The technique is optimized for detecting the dipolar interaction between nitroxides, which are usually added in the form of methane thiosulfonate spin labels (MTSSL) via site-directed mutagenesis.^13^ Trityl radicals^14,15^ and metal centers such as Gd(II),^16^ Mn(II),^17^ and Cu(II)^18,19^ are also emerging as alternative spin labels with controllable spectroscopic properties.

Despite the versatility of spin labels, the search for alternative probes, such as endogenous paramagnetic centers that eliminate the need to incorporate a non-native group in the macromolecule, is an active area of research.^20−24^ Furthermore, in contrast to spin labels attached at surface accessible sites, native groups are rigidly held within protein structures. As a consequence, distance and orientation distributions are narrower, enabling a more accurate analysis of these parameters. In particular, orientational analysis of PDS traces can be used to orientate interacting proteins or protein subunits with respect to one another.^25^ Traditionally, the research of native paramagnetic probes has been mainly focused on metal-based centers such as Cu(II),^26,27^ low-spin and high-spin Fe(III),^28−30^ iron sulfur clusters,^25,31,32^ and manganese clusters.^33^ The sensitivity of PDS experiments on metal centers can suffer from the small fraction of spins within the excitation bandwidth of a typical rectangular microwave pulse. However, at the same time the orientation selection can provide valuable structural information.

The photoexcited triplet states of organic chromophores have been proposed as a probe for PDS.^34−40^ Many biological molecules, such as photosynthetic proteins,^41,42^ heme-proteins,^36,43^ and flavoproteins,^44^ contain moieties that can act as optically activated spin centers. They are closed shell in their ground state but form electron spin-active triplet states after photoexcitation at appropriate wavelength. In addition to being photoactivated, allowing switching between spin-active and spin-silent states, these probes are formed in a spin-polarized state and thus their ESR signals are stronger compared to those of Boltzmann populated paramagnetic centers.^45,46^

When the triplet states are sufficiently long-lived (microsecond or millisecond time scale) and formed with high yield, they can be measured using time-resolved or pulsed ESR coupled to laser excitation. Triplet state ESR provides relevant spectroscopic information due to orientation selection:^41,47−49^ in hyperfine spectroscopy the relative orientation of the hyperfine tensors to the zero-field splitting (ZFS) tensor within the molecular structure can be extracted and validated with DFT calcualtions.^48^

Among the triplet state pulsed ESR techniques, light-induced DEER (LiDEER)^34,35^ and laser-induced magnetic dipolar (LaserIMD) spectroscopy^50^ are two PDS techniques that allow the dipolar coupling between a photoexcited triplet and a stable radical to be studied. Both use a laser pulse to generate the triplet state and microwave pulses to manipulate the spins. Comparisons between the two techniques have previously been carried out both at X-band and at Q-band.^51,52^ LiDEER uses laser photoexcitation introduced at the beginning of the sequence to allow the triplet state to act as the detection spin and the electron spin polarization is exploited to enhance the signal. In LaserIMD, the dipolar modulation is induced by optically switching on the triplet state while the signal of the observer spin is detected. The detection sequence can use either a primary Hahn echo sequence^50^ or a refocused echo sequence (ReLaserIMD),^36^ where ReLaserIMD yields a symmetric zero time facilitating accurate determination of this time point. The investigation on a porphyrin-based spectroscopic ruler set the accessible distance range between 1.5 and 8 nm.^35^

Recently, we introduced a new triplet-based PDS technique, light-induced triplet–triplet electron resonance spectroscopy (LITTER), which measures the dipolar interaction between two photoexcited triplet states.^53^ The benefits of the LITTER experiment were evidenced, and significant orientation selection was demonstrated for a model diporphyrin–peptide system. In the LITTER experiment, as with all one-dimensional (1D) PDS techniques, multiple traces must be recorded at different fields in order to sample the orientation selection fully. For single-frequency PDS techniques such as relaxation-induced dipolar modulation enhancement (RIDME)^54^ and the single-frequency technique for refocusing dipolar couplings (SIFTER),^55^ it has been shown that it is possible to use shaped microwave pulses to extract the complete orientation dependence of the dipolar spectrum in a single two-dimensional (2D) frequency-correlated (FC) experiment.^56−58^ The same principle can be applied to light-induced single-frequency PDS techniques.

In this work, we demonstrate the first example of a two-dimensional light-induced frequency-correlated PDS experiment: FC ReLaserIMD. Shaped microwave pulses were used to excite the full nitroxide spectrum allowing the detection of the complete orientation dependence of the experiment to be determined in a single pulse sequence. To verify these results, we also present orientation-selective ReLaserIMD. These two experiments utilize the anisotropy of the nitroxide spectrum at Q-band. Finally, orientation-selective LiDEER experiments, which exploit both the anisotropy of the triplet state and the nitroxide spectra, were used to complete the orientational analysis.

A geometrically well-defined model compound, the bis-labeled peptide [**1**] (see Figure 1 for the amino acid sequence and Figures 2c and S5 for the molecular structure), was chosen for these proof-of-concept experiments, as a narrow distance distribution between the paramagnetic centers, connected by a rigid α-helical linker, was demonstrated in previous light-induced PDS investigations.^34,35^ The results of the orientation-selective data sets, recorded with the three different techniques, are compared and synergistically modeled using orientation-dependent simulations to prove the potential of light-induced PDS techniques in providing accurate information on the relative position of the spin-active moieties.

**Figure 1:**
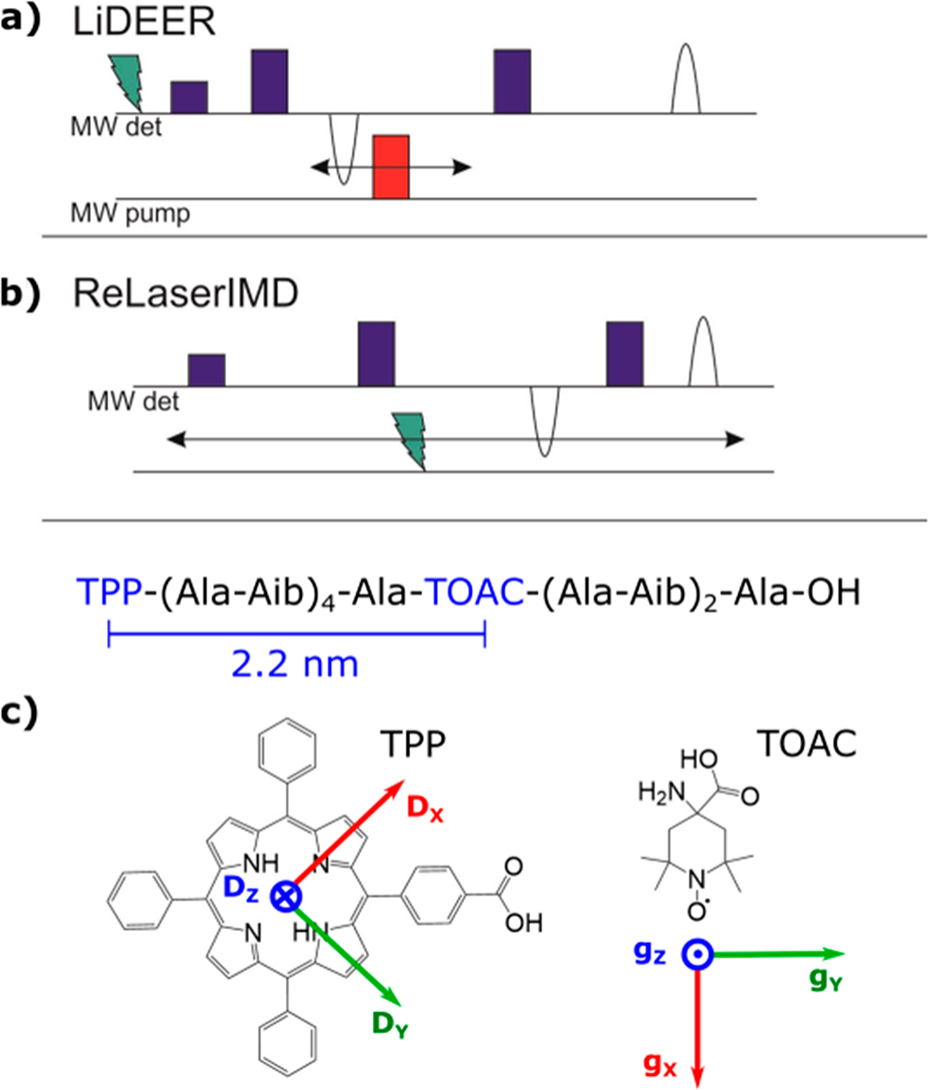
(a) Light-induced double electron–electron resonance (LiDEER) pulse sequence. (b) Refocused Laser-induced magnetic dipole (ReLaserIMD) spectroscopy pulse sequence. (c) Amino acid sequence of molecule [**1**]. Key: TPP (5(4′-carboxyphenyl)-*10*,*15*,*20*-triphenylporphyrin), Ala (l-alanine), Aib (α-aminoisobutyric acid), and TOAC (2,2,6,6-tetramethylpiperidine-1-oxyl-4-amino-4-carboxylic acid). The distance between the center of the porphyrin and the midpoint of the N–O bond in TOAC as predicted by DFT is indicated. The chemical structures of TPP and TOAC are included, showing their respective ZFS tensor frame^59,60^ (TPP triplet, left) and g tensor frame^61^ (nitroxide radical in TOAC, right).

**Figure 2:**
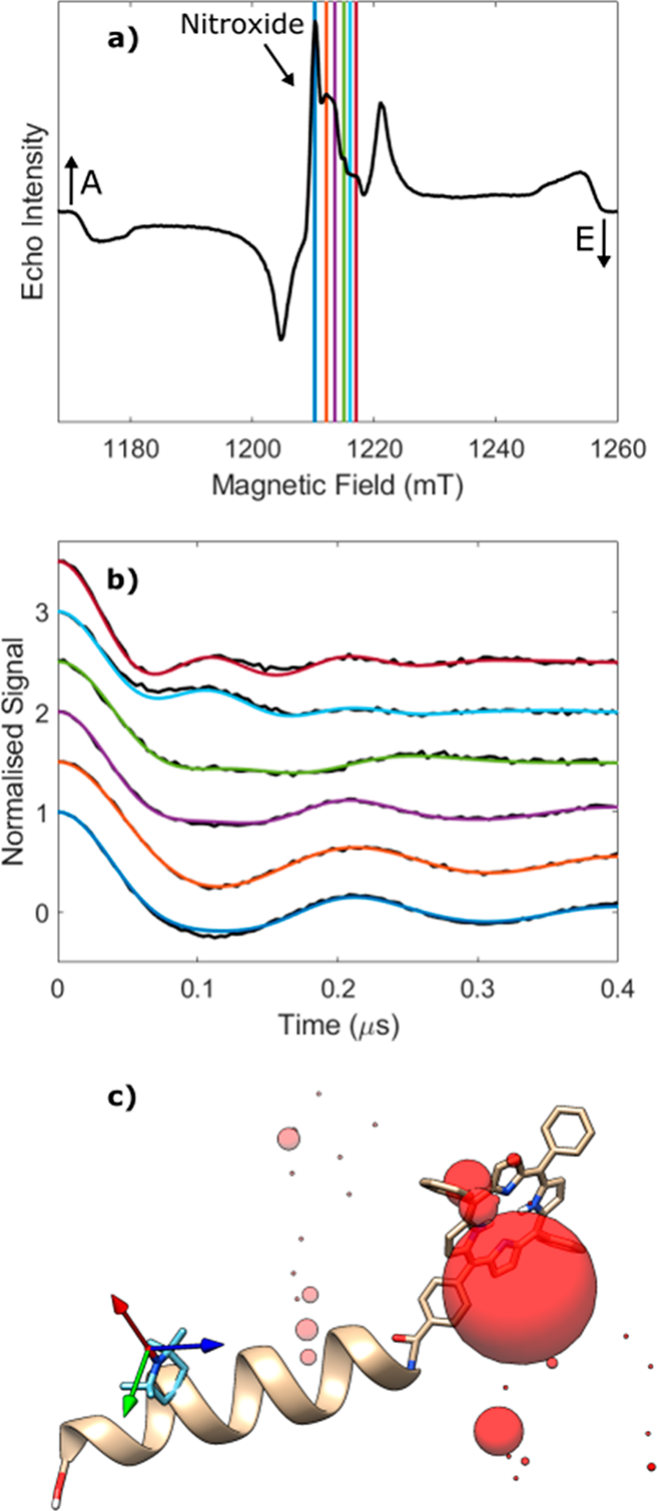
(a) Electron spin echo field swept spectrum of [**1**] measured using parameters optimized for the nitroxide radical. The experimental field values used for ReLaserIMD are indicated as vertical lines. (b) Measured ReLaserIMD traces (black) shown with the orientation-dependent simulations (colors correspond to field positions in panel a). The modulation depth has been normalized to 1 for ease of analysis. (c) DFT-optimized structure of molecule [**1**] showing the different positions of the porphyrin center determined by the fitting procedure as colored spheres, relative to the nitroxide g tensor frame (arrows: red = g_*x*_, green = g_*y*_, blue = g_*z*_). The diameter of the sphere is proportional to the number of times a single porphyrin position contributes to the complete fit shown in panel b. The experimental conditions and the parameters of the data analysis are reported in the Supporting Information.

## Orientation Selection in ReLaserIMD

Orientation-resolved ReLaserIMD data were obtained by measuring a series of dipolar evolution time traces at different field positions covering the complete nitroxide spectrum of the model peptide (Figure 2a,b). The field-swept electron spin echo spectrum of the bis-labeled peptide shows the narrow central absorption signal due to the TOAC spin label and the broad contribution due to the TPP triplet state with an *eaeaea* spin polarization pattern (e = emission, a = enhanced absorption). The PDS form factors show a significant orientation selection as the pump pulse is moved across the ESR spectrum leading to pronounced differences in the dipolar frequency ω_dd_ with field position. The variation in ω_dd_ indicates a strong correlation between the dipolar interspin vector with respect to the nitroxide moiety. This is as expected for the model system where the TOAC moiety holds the nitroxide in a rigid position with respect to the peptide backbone and the stiff long axis of the peptide limits the orientation flexibility of the interspin vector with respect to it.

The ReLaserIMD technique is particularly useful in this work as it allows more accurate selection of the experimental zero time, compared to Hahn echo detected LaserIMD.^36^ This is vital to the interpretation of different frequency components within the PDS trace shape found in orientation-selective data sets.

The ReLaserIMD traces recorded at different field positions all yielded similar modulation depths. This likely indicates either that the laser power used was sufficient to saturate the transition and excite both Q_*x*_ and Q_*y*_, or that phototautomerism of the indole protons exchanges the Q_*x*_ and Q_*y*_ by a pseudorotation, making the directions of the transition dipole moment indistinguishable in the porphyrin plane.^60^ Furthermore, no magneto-photoselection effects were recorded under the conditions of these experiments as minimal differences were seen in both field sweep spectra or ReLaserIMD traces recorded with the laser polarization horizontal and vertical to B_0_ (see Figure S3). Because the optical transition frame aligns with the ZFS splitting frame generated in the triplet state, as the Q_*x*_ and Q_*y*_ are interchangeable, the correlation between the g tensor of the nitroxide and the ZFS frame is lost. For this reason, while the information on the orientation of the spin–spin vector relative to the g tensor reference frame of the nitroxide can be extracted precisely, no information on the mutual orientation of the two paramagnetic centers is available from this experiment.

Orientation-dependent simulations were performed using the algorithm previously published,^8^ and the results were fitted to the experimental results using an iterative least-squares fitting process, similar to that reported here.^53,62^ As it is not possible to resolve the orientation of the ZFS tensor from these experiments, in the ReLaserIMD simulations the TPP moiety was modeled as a single point of spin-density located at the center of the porphyrin moiety. In the fitting process the modulation depth was normalized to 1 to account for variations in the measured modulation depths, which may arise from small oscillations in laser power during the experimental acquisition.

The results of these simulations agree well with the density functional theory (DFT) calculations (details are reported in the Supporting Information). The rigidity of the system is demonstrated by the presence of a single dominant conformer, which corresponds to an energy minimum where the backbone carboxylate group is axial to the piperidinyl ring of TOAC as depicted in Figure 2c. The simulations indicated that a small amount of a second chair conformation, with the backbone carboxylate group equatorial to the ring may also be present in a lower proportion together with the twist boat conformation (see Figure S7). These conclusions are perfectly in line with X-ray diffraction on an analogous peptide where the porphyrin moiety was not covalently bound.^63^

## Frequency-Correlated ReLaserIMD

**Figure 3:**
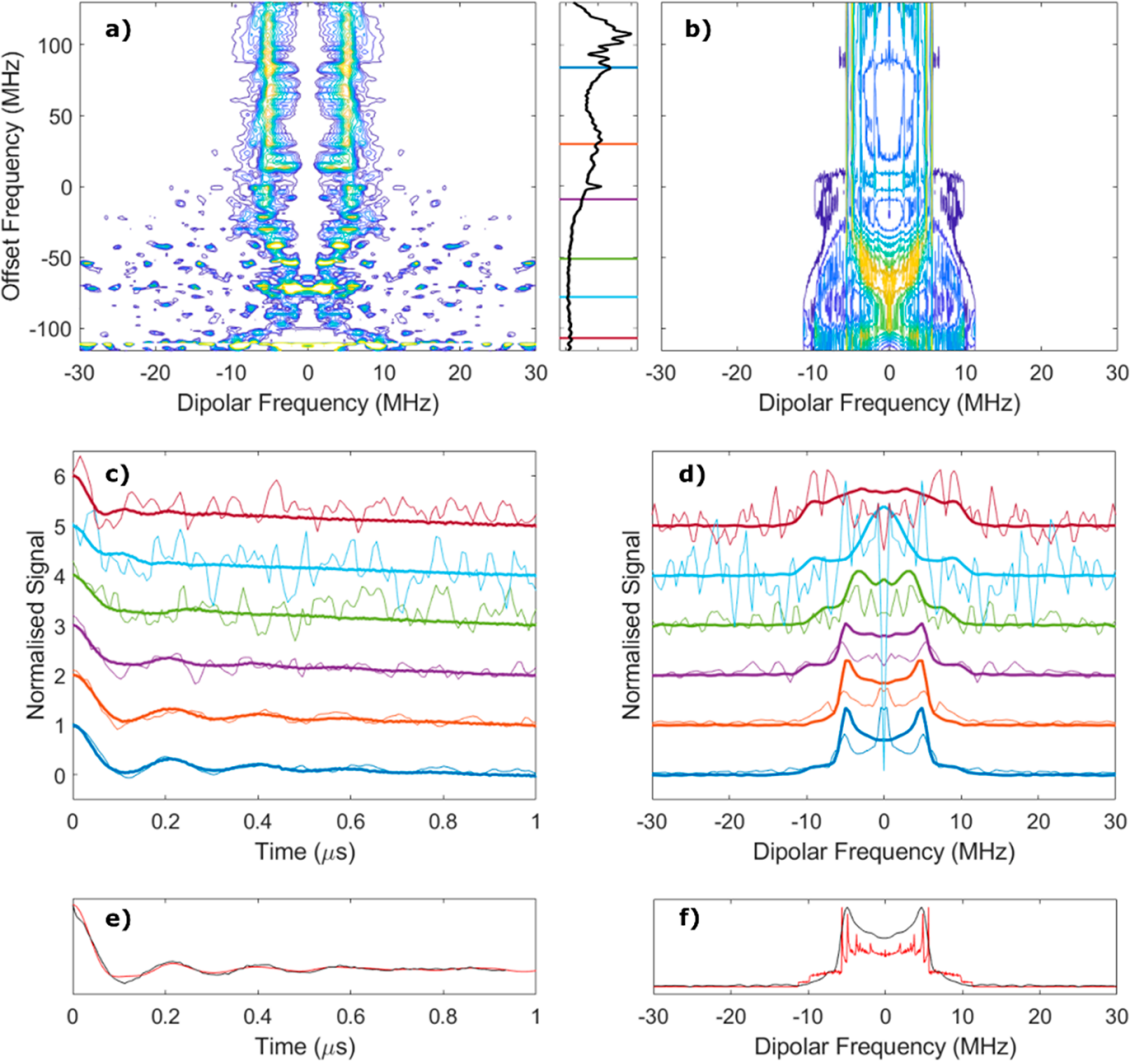
(a) Complete normalized experimental FC ReLaserIMD spectrum of [**1**] and (b) corresponding simulation with the panel between panels a and b displaying the Fourier transform of the FC ReLaserIMD echo recorded at the zero time of the experiment, with the experimental frequency values used for the ReLaserIMD indicated as horizontal lines. (c) Time domain slices of the FC ReLaserIMD (thin lines) plotted with the orientation-selective ReLaserIMD traces recorded at equivalent fields (thick lines). (d) Frequency domain slices of the FC ReLaserIMD (thin lines) plotted with the orientation-selective ReLaserIMD results recorded at equivalent fields (thick lines). The colors in panels c and d correspond to the frequency and field positions indicated in Figure 2a and the panel between panels a and b. (e) Sum over the offset frequency dimension of the FC ReLaserIMD experimental data shown in black and simulation shown in red. (f) Fourier transform of panel e. The experimental conditions and the parameters of the data analysis are reported in the Supporting Information.

In order to obtain the full orientation information from PDS data, we have developed FC ReLaserIMD. We have modified the ReLaserIMD sequence to use hyperbolic shaped pulses, which allow the bandwidth of the pulse to be extended compared to rectangular pulses and make complete excitation of the nitroxide spectrum at Q-band possible. The echo transients were collected for each time step of the laser flash, and after background correction along the ReLaserIMD time dimension, the absolute of the Fourier transform was calculated. Results of this experiment conducted on molecule [**1**] are presented in Figure 3a.

Simulations of the two-dimensional (2D) FC ReLaserIMD, carried out using the conformer orientations found from fitting the individually measured one-dimensional (1D) ReLaserIMD traces, reproduced the experimentally measured orientational features of the FC ReLaserIMD experiment (Figure 3b). Good agreement was also seen between the experimental orientation-selective 1D ReLaserIMD form factor traces with their Fourier transforms and the corresponding slices of the 2D FC data set in the same spectral regions (Figure 3c,d). In this way, we prove that the orientation correlation between the spin–spin vector and the nitroxide molecular frame can be accessed in a single experiment, whereas 1D techniques require a combination of multiple experiments. The orientation distribution fitting is the same for both data sets.

Looking at the results from a different perspective, orientation selection is informative, but it significantly complicates experiments and data analysis if only the distance distribution is of interest. The presence of orientation selection in the 1D ReLaserIMD experiments means that analysis of a single trace with *DeerAnalysis*,^64^ which uses an orientation-independent kernel function, may yield inaccurate distance distribution results. When the orientation selection is solely due to the nitroxide moiety, it may be possible to remove this selectivity by measuring and combining several ReLaserIMD or LiDEER traces at different fields and/or detection-pump offsets.^58^ However, the number and weighting of such traces required is not easily determined, and in the case of LiDEER the bandwidth of the resonator will restrict the experimental offsets that can be used between pump and detection frequencies (see following section), preventing the complete sampling of all orientation information. By comparison, orientation-averaged dipolar spectra can be easily obtained from the 2D experiment by summing the entire offset frequency domain to yield the complete Pake pattern of the dipolar spectrum (Figure 3e,f). The distance distribution, yielded from analysis of this averaged trace using *DeerAnalysis*, is presented in the Supporting Information (Figure S9).

## Orientation-Selective LiDEER

Although the orientation-selective ReLaserIMD experiments allowed the relative spatial position of the center of the TPP moiety with respect to the TOAC nitroxide g tensor frame to be determined, the technique did not yield information on the relative orientations of the two paramagnetic moieties, as there was no orientation dependence in the light excitation of the porphyrin. The LiDEER traces encode additional information on the geometrical arrangement of the two spin labels, performing orientation selection not only with the detection pulse sequence but also with the microwave pump pulse. The relative orientation of the nitroxide g tensor frame and the porphyrin ZFS tensor frame can be derived. Combining this with the information on both tensor directions in the individual moiety molecular frames, which is accessible in the case of the nitroxide–porphyrin pair,^59−61^ the relative orientation of the two paramagnetic centers can be ultimately derived together with the relative spatial position, as determined from 1D and 2D FC ReLaserIMD.

**Figure 4:**
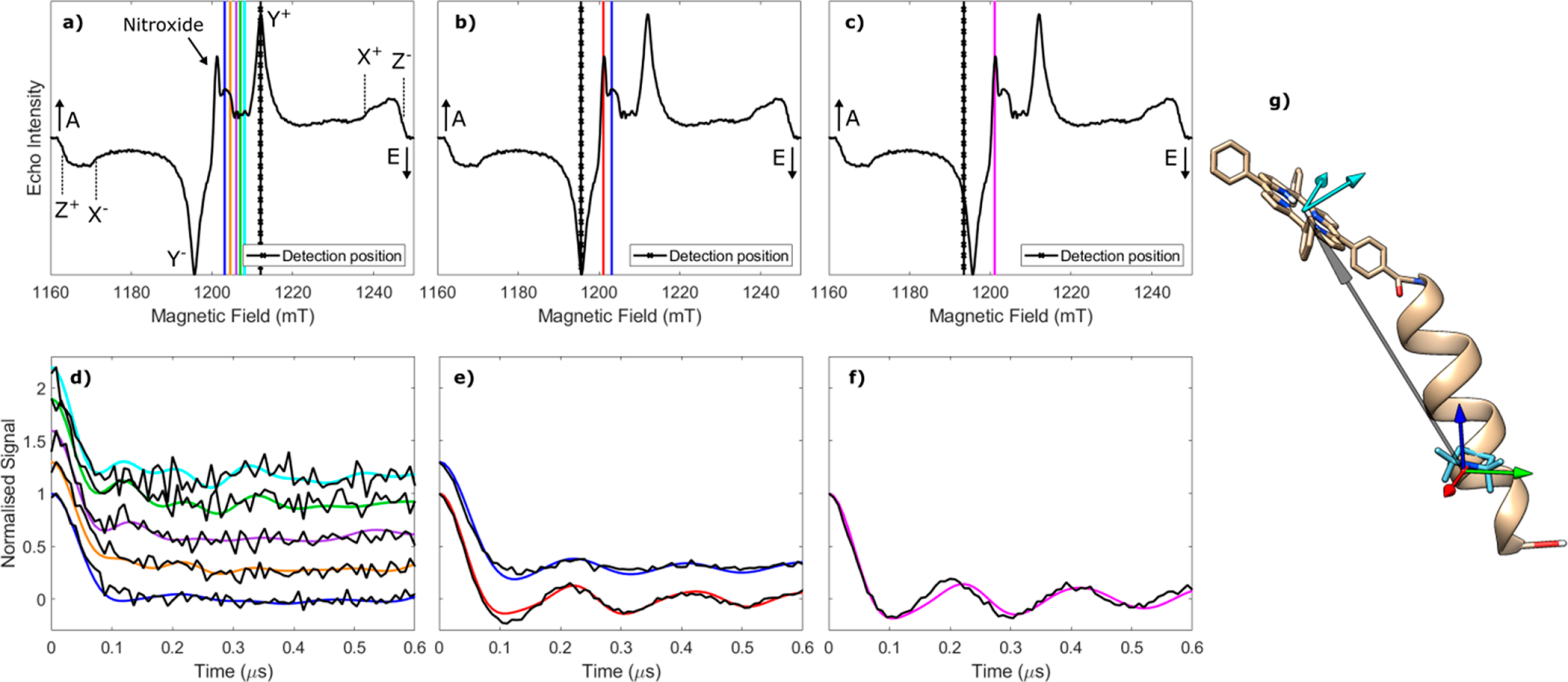
(a–c) Electron spin echo field swept spectra showing the detection (black crossed vertical lines) and pump (colored vertical lines) positions used for LiDEER. (d–f) Experimental (black) and simulated (colored) LiDEER traces. The colors of the simulated traces correspond to the pump positions shown in panels a–c. (g) DFT-optimized structure of molecule [**1**] showing the positions and *z* axes of the ZFS tensors of the porphyrin centers (cyan arrow) most contributing to the best fitting set of simulated LiDEER traces, relative to the nitroxide g tensor frame (arrows: red = g_*x*_, green = g_*y*_, blue = g_*z*_) and the spin–spin vector (gray arrow). The experimental conditions and the parameters of the data analysis are reported in the Supporting Information.

To this end, LiDEER experiments using a refocused Hahn echo detection scheme were performed with variable offsets between pump and detection frequencies and variable fields as reported in Figure 4. In all DEER experiments the maximum frequency offset between pump and detection pulses is limited by the bandwidth of the resonator. For this reason, it was necessary to record three sets of LiDEER experiments to sample the orientation selection of both the nitroxide and the triplet state. Initially, a set of five experiments, in which the detection frequency and field were fixed to the maximum positive peak in the triplet spectrum and the pump position varied across the nitroxide spectrum, were conducted (Figure 4a,d). A second set of two experiments, in which the detection frequency and field were fixed to the maximum negative peak in the triplet spectrum and the pump position varied across the nitroxide spectrum, were completed (Figure 4b,e).

Pumping at the position corresponding to the blue line in Figure 4a,b and detecting either at the minimum or at the maximum of the triplet spectrum, corresponding to the Y canonical orientation of the ZFS tensor, yielded traces with similar shape within signal-to-noise (blue traces Figure 4d,e). This was as expected, because the two maxima of the triplet spectra derive from resonance of the same molecular orientation.

The large width of the triplet spectrum made it impossible within the resonator bandwidth to sample all orientations of the triplet with respect to the external magnetic field while pumping the nitroxide spin. Although a complete set of experiments was precluded, a final trace was recorded where the detection frequency was selected to sample a field position of the triplet spectrum sampling orientations intermediate between those corresponding to the X and Y canonical orientations (Figure 4c,f). The pump frequency was chosen to be resonant with the maximum of the nitroxide spectrum. Comparing this to the trace recorded with the detection pulses resonant with the Y transition, while using the same pump pulse position, showed a difference in oscillation frequency between the traces. This indicates some orientation selection from the detection pulses, limited by the small difference between the detection positions.

Using the dominant orientation of the dipolar vector with respect to the g tensor of the nitroxide determined from the ReLaserIMD experiments, traces corresponding to a sample of all possible relative orientations of the nitroxide g tensor frame and the porphyrin ZFS tensor frame were calculated. The ZFS principal axes (Figure 1c) and the spin density of the porphyrin triplet state were calculated using DFT (Figure S5) and are in excellent agreement with experimentally determined examples.^65^

In order to simultaneously analyze the complete set of orientation-dependent LiDEER traces and to account for the changing efficiency in the pump pulse when it is positioned at different frequencies within the resonator bandwidth, the modulation depth was normalized to 1 for both the simulated and experimentally determined LiDEER form factors. The normalized experimental form factors were fitted to the calculated library of traces using an iterative process, where the number of iterations matched the number of contributions of the dominant conformer to the fit obtained for the ReLaserIMD data sets (Figure 2). The ZFS *Z*-axis of the two dominant conformers, obtained from this fitting process, are presented in Figure 4g; the resulting orientations of the ZFS tensor in the molecular frame show good agreement with the energy-minimized DFT structure and lie within the expected flexibility of molecule [**1**]. An improvement to the fit might be gained by allowing flexibility in both the position and orientation of the two centers due to the conformational distribution expected, as the PDS experiments were performed at cryogenic temperatures on frozen solutions (see distance distribution in Figure S9). However, this increases the parameter space, and therefore, the uniqueness of a fit is likely reduced.

In conclusion, we have demonstrated that orientation selection effects can be observed in both ReLaserIMD and LiDEER. We have completed a comprehensive analysis of the results, exploiting the complementary information provided by the two techniques, to localize the nitroxide center with respect to the TPP moiety, yielding atomic-level geometric structural information, and validated these results using DFT calculations. The combination of the two techniques enables the parameter space required for simulation to be reduced, simplifying the simulation process while enabling the relative positions and orientation of the centers to be accurately determined.

Additionally, we have presented the first example of 2D FC ReLaserIMD with a nitroxide observer sequence based on hyperbolic pulses, achieving, for the first time, complete excitation of a nitroxide spectrum at Q-band on a commercially available ESR spectrometer. This technique leads to an improved frequency resolution and a larger span of frequency offsets compared to orientation-selective ReLaserIMD experiments in a reduced experimental measurement time. Alternatively, summation of the experimental results of FC ReLaserIMD over the offset frequency domain yields an orientation-averaged dipolar trace enabling orientation-independent analysis.

Rigidly embedded endogenous spin-centers, such as native cofactors which are either present in a paramagnetic state or can be photoexcited to a suitable triplet state, are of significant interest for the measurement of PDS traces on unmodified protein systems. There is particular interest in the use of these centers in near-native environments where conventional spin labels, such as nitroxides, may be denatured.^66^ While the rigidity of the binding site within the protein structure may result in narrow distance distributions enabling more accurate reporting of protein structure changes, the likely orientation selection of PDS experiments on such systems necessitates careful analysis. Consequently, the combined approaches presented here for the PDS experiments measured between an optically activated probe and a permeant paramagnetic center will likely prove invaluable in the analysis of endogenous PDS EPR studies. These methods offer the ability either to obtain geometric information on the structure of macromolecules, beyond the distance distribution, or to reduce the complexity of the measurements such that orientation-independent analysis of the dipolar trace can be confidently performed. In a previous study we demonstrated the feasibility of light-induced PDS on heme-proteins.^36^ The next step is to apply the orientation-resolved techniques to gain further structural information, which is biologically relevant, on proteins containing bound optically active cofactors.

## Methods

The experimental and computational methods are described in the Supporting Information.

## Supporting Information Available

Experimental methods (sample preparation and ESR spectroscopy), computational methods (density functional theory calculations and orientation-dependent simulations), and results (spectroscopic characterization of molecule [**1**], DFT results, orientation-selective ReLaserIMD, frequency-correlated ReLaserIMD, orientation-selective LiDEER) (PDF)
