# Supplementary material for: Orientation-Selective and Frequency-Correlated Light-Induced Pulsed Dipolar Spectroscopy
Source: J Phys Chem Lett. 2021 Apr 15;12(15):3819–26. doi: 10.1021/acs.jpclett.1c00595 (PMC8154851; doi:10.1021/acs.jpclett.1c00595)
Supplement: Supplementary file 1 — jz1c00595_si_001.pdf [file jz1c00595_si_001.pdf]

Supporting Information for

Orientation-Selective and Frequency-Correlated Light-Induced Pulsed Dipolar Spectroscopy

Alice M. Bowen,[1,2] Arnau Bertran,[2] Kevin B. Henbest,[2] Marina Gobbo,[3] Christiane R. Timmel[2] and Marilena Di Valentin*[3]*

[1] *Department of Chemistry, Photon Science Institute and The National EPR Research Facility, The University of Manchester, Oxford Road, Manchester M13 9PL, United Kingdom.*

[2] *Center for Advanced Electron Spin Resonance and Inorganic Chemistry Laboratory, Department of Chemistry, University of Oxford, South Parks Road, Oxford OX1 3QR, United Kingdom.*

[3] *Department of Chemical Sciences, University of Padova, Via Marzolo 1, 35131 Padova, Italy.*

* Corresponding authors: alice.bowen@manchester.ac.uk, marilena.divalentin@unipd.it

CONTENTS

S1. Experimental methods

S1.1. Sample preparation

S1.2. ESR spectroscopy

S2. Computational methods

S2.1. DFT calculations

S2.2. Orientation-dependent simulations

S3. Results

S3.1. Spectroscopic characterization of molecule [1]

S3.2. DFT results

S3.3. Orientation-selective ReLaserIMD

S3.4. Frequency-correlated ReLaserIMD

S3.5. Orientation-selective LiDEER

S1. EXPERIMENTAL METHODS

S1.1. Sample preparation

Molecule **[1]** was synthesized as reported by Di Valentin *et al.*[1] Samples for ESR were prepared to 100 µM in 98% deuterated methanol, 2% D_2O. Samples were degassed by several freeze-pump-thaw cycles, sealed and frozen in liquid nitrogen prior to insertion into the spectrometer.

S1.2. ESR spectroscopy

ReLaserIMD and LiDEER experiments were conducted in Elexsys E580 and E680 spectrometers (Bruker). The E580 was fitted with a spinjet Arbitrary Waveform Generator (AWG). Experiments used a TII resonator at Q-band (34 GHz). The temperature was maintained at 20 K using liquid helium and a CF935 cryostat (Oxford Instruments) with an ITC103 temperature controller (Oxford Instruments). Laser excitation was provided by an OPO pumped by the third harmonic of a Nd:YAG laser (Opotek, Opolette355) operated at a repetition rate of 20 Hz (5 ns pulses) at a wavelength of 512 nm, with energy of ca. 2.5 mJ per pulse.

LiDEER measurements used rectangular 40 ns pump pulses and 40 ns detection pulses and time delays τ_1 = 120 ns and τ_2 = 1000 ns (Figure S.1a). ReLaserIMD (refocused echo three-pulse) used rectangular $\pi/2$ = 28 ns and $\pi/2$ = 28 ns pulses and time delays τ_1 = 1200 ns and τ_2 = 200 ns (Figure S.1b). FC ReLaserIMD used the same pulse sequence ReLaserIMD, with shaped pulses generated by the AWG (Figure S.1c). The pulses used were hyperbolic with a sech/tanh frequency function and a WURST amplitude function calculated to have bandwidths of ca. 300 MHz, B_1 fields of 10 MHz, measured from the TII resonator, and lengths of 800 ns (π) and 1600 ns ($\pi/2$) were calculated according to previous work.[2] Using π pulses with double the chirp rate of the $\pi/2$ pulses allows all

frequency components of the primary echo to be refocused at the same time. This gives a constant zero time of all components relative to the laser flash. Application of the 2nd π pulse causes the zero times of the different frequency components to vary with the time at which each component is inverted by the 2nd π pulse, however this is predictable and the zero times can easily be corrected to occur at the same time.

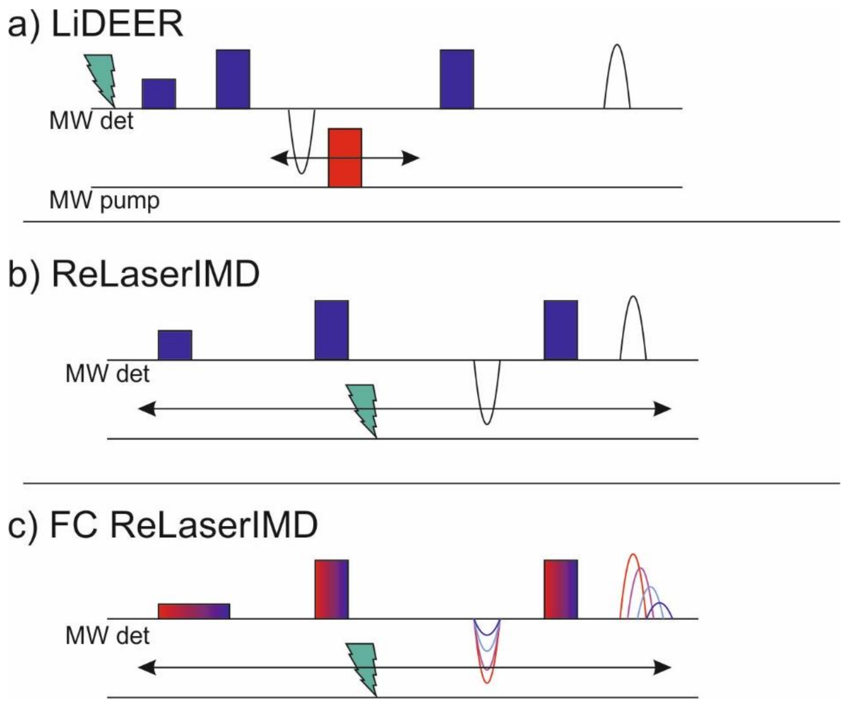

Figure S1. ESR pulse sequences for (a) LiDEER, (b) ReLaserIMD and (c) FC ReLaserIMD.

The signal to noise ratio (SNR) of the dipolar traces is defined as the ratio between the modulation amplitude and the noise level. The experimentally observed SNR is more favourable for 1D LaserIMD compared to 2D FC LaserIMd and LiDEER. The reasons for this are as follows. Regarding the LiDEER vs ReLaserIMD, comparisons between the two techniques have previously been carried out both at X-band and at Q-band.[3] In general, at Q-band LaserIMD recorded on the nitroxide signal maximum yields a higher SNR than LiDEER at short dipolar evolution times (around 2 μs), even when the LiDEER pump pulse is set resonant with the maximum of the nitroxide and the detection pulses are resonant with the maximum of the triplet

signal. In the series of LiDEER experiments conducted here, the pump pulse position is changed to be resonant with different parts of the nitroxide spectrum. When this pulse is not resonant with the nitroxide signal maximum, the modulation depth is reduced compared to traces where this is the case and consequently the SNR is reduced. The LiDEER data are all plotted with the modulation depths normalized to one, which shows how SNR changes with different combinations of pump and detection pulses. Conversely, the ReLaserIMD traces recorded at different field positions all yielded similar modulation depths and as such have similar SNRs.

When comparing the 2D FC LaserIMD with the corresponding 1D LaserIMD data set, the worse SNR for the 2D FC LaserIMD data can be partially ascribed to the different recording time for a 2D experiment compared to a 1D experiment. Recording the 2D data required the echo transient to be stored which takes significantly longer than recording the integral of the echo intensity in the Bruker Xepr software. A reduced number of scans is therefore accessible for 2D FC LaserIMD. Secondly, in the 2D experiment the same video gain and number of scans must be used for recording at all offset frequencies. This means that the SNR of the data recorded on the nitroxide g_z of the spectrum, where the echo signal detected is lower intensity, is necessarily worse than the traces on the nitroxide signal maximum since all of the 2D traces are recorded with the same settings in one single experiment.

Details of the field and frequency experiments for the ReLaserIMD and LiDEER are listed in Tables S1 and S2, respectively.

Table S1. Field positions and frequencies used in the orientation selective ReLaserIMD

Frequency (GHz)	Field (mT)
**33.998**	1210.2
**33.998**	1212.1
**33.998**	1213.5
**33.998**	1215.0
**33.998**	1216.0
**33.998**	1217.0

Table S2. Field positions and frequencies used in the orientation selective LiDEER experiment.

Detection Frequency (GHz)	Pump Frequency (GHz)	Field (mT)
**33.810**	34.060	1211.8
**33.810**	34.021	1211.8
**33.810**	33.979	1211.8
**33.810**	33.951	1211.8
**33.810**	33.922	1211.8
**34.038**	33.882	1213.7
34.038	33.824	1213.7
34.038	33.824	1201.9

Electron spin-echo (ESE) experiments (i.e. field sweeps, T_2 and delay after flash (DAF) experiments) were performed in an over-coupled resonator using a standard Hahn echo sequence preceded by a laser flash (laser flash – DAF – $\pi/2 - \tau - \pi - \tau$ – echo), with a pulse lengths of π = 40 ns and $\pi/2$ = 20 ns. Field swept electron spin-echo spectra were recorded using a DAF of 1600 ns, a τ value of 200 ns, a laser energy of 2.5 mJ per flash. Triplet state parameters were extracted via simulation of the spectrum using the Matlab® *EasySpin* routine (*pepper* function).[4]

T_2 experiments were recorded on the canonical Y^- and Y^+ positions of the porphyrin triplet spectrum and on the nitroxide maximum with the laser both on and off. Delay after flash (DAF) experiments were recorded on the canonical Y^- and Y^+ positions of the porphyrin triplet spectrum.

Field sweeps were recorded with light polarized both vertical and horizontal to the magnetic field. Under the circumstances of this experiment, little magneto-photoselection was observed as the laser conditions used caused saturation of the transition (Figure S4).

S2. COMPUTATIONAL METHODS

S2.1. DFT calculations

Initial geometries for molecule **[1]** were built based on previous DFT calculations[5] and XRD studies,[6] using UCSF Chimera.[7] Geometry optimizations and spin density calculations were performed *in vacuo* using Gaussian® 09 (revision A.02).[8] Ground state geometry optimizations were carried out using the PBE1PBE functional and the 6-31g(d) basis set. The geometry of the TPP moiety was subsequently optimized in the triplet state using the functional B3LYP and the basis set SV(P). Electronic spin densities were obtained by single-point calculations on the previously optimized structures, using the functional B3LYP and the basis set EPR-II.[9] The Zero-Field Splitting (ZFS) tensor orientation of the porphyrin triplet and the g tensor orientation of the nitroxide radical were calculated using Orca (release version 4.2.0),[10] with the functional B3LYP and the basis set EPR-II. The spin-spin contribution to the ZFS was calculated using computed UNO (spin-unrestricted natural orbital) determinants.[11]

S2.2. Orientation-dependent simulations

A protocol adapted from that described by Lovett *et al.*,[12] into which the ZFS tensor of the triplet was also incorporated, was used to simulate both the orientation selective LiDEER and ReLaserIMD experiments. The frames of reference used are shown in Figure 1.

In the case of the ReLaserIMD experiments, the bandwidth of the pump pulse was set to be infinite, reflecting the fact that the light pulse used in these experiments was non-selective. A library of pre-simulated ReLaserIMD traces was fitted to the experimental data following the protocol of Marko *et al.*[13] 200 least squares fitting iterations were performed in which the trace providing the best improvement in fit to the experimental data was added to the previous best fits. This simulation allowed the relative position of the porphyrin triplet and nitroxide to be determined, but did not provide information on the orientation of the ZFS tensor on the porphyrin. The best fitting relative position of the two centers from the ReLaserIMD simulation was used as a starting point for the LiDEER simulation which allowed the orientation of the ZFS tensor on the porphyrin to be determined. The number of least squares fitting iterations matched the number of contributions of the dominant conformer to the fit obtained for the ReLaserIMD data sets.

In all cases the following parameters were used to simulate the porphyrin triplet state spectra: D = 41.4 mT, E = -8.4 mT, H-strain = 3.8 mT, g = 2.0059, linewidth = 0.7 mT and triplet state sublevel populations $p_x = 0.32$, $p_y = 0.46$ and $p_z = 0.23$. The nitroxide was simulated using the following parameters: g = [2.0101, 2.0071, 2.0034], A_N = [0.4573, 0.7019, 3.6418] mT, H-strain = [0.5636, 0.4955, 0.7120] mT.

S3. RESULTS

S3.1. Spectroscopic characterization of molecule [1]

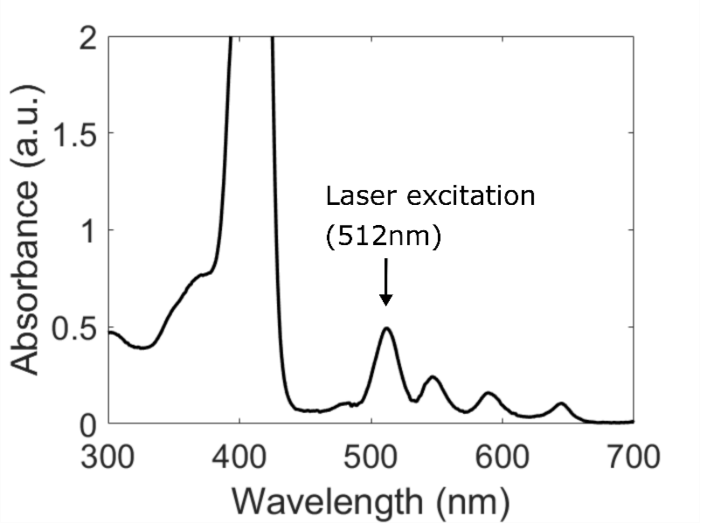

Figure S2. UV-Vis absorption spectrum of 100 μM **[1]** in a mixture of 98% d_4-methanol and 2% D_2O at room temperature. The laser excitation wavelength used for the ESR experiments, corresponding to the most intense maximum of the Q-band region, is indicated. Spectrum measured using a Cary60 UV-Vis spectrometer (Agilent) and a 3.00 mm quartz ESR tube.

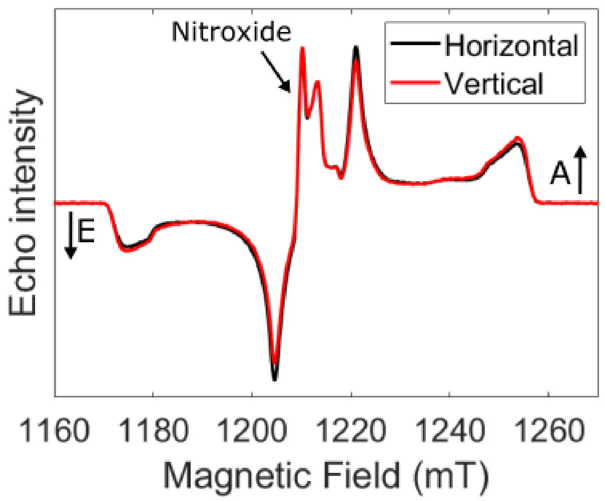

Figure S3. Electron spin echo field swept spectra of [**1**] after photoexcitation at 512 nm with polarized light parallel (black) and perpendicular (red) to the external magnetic field.

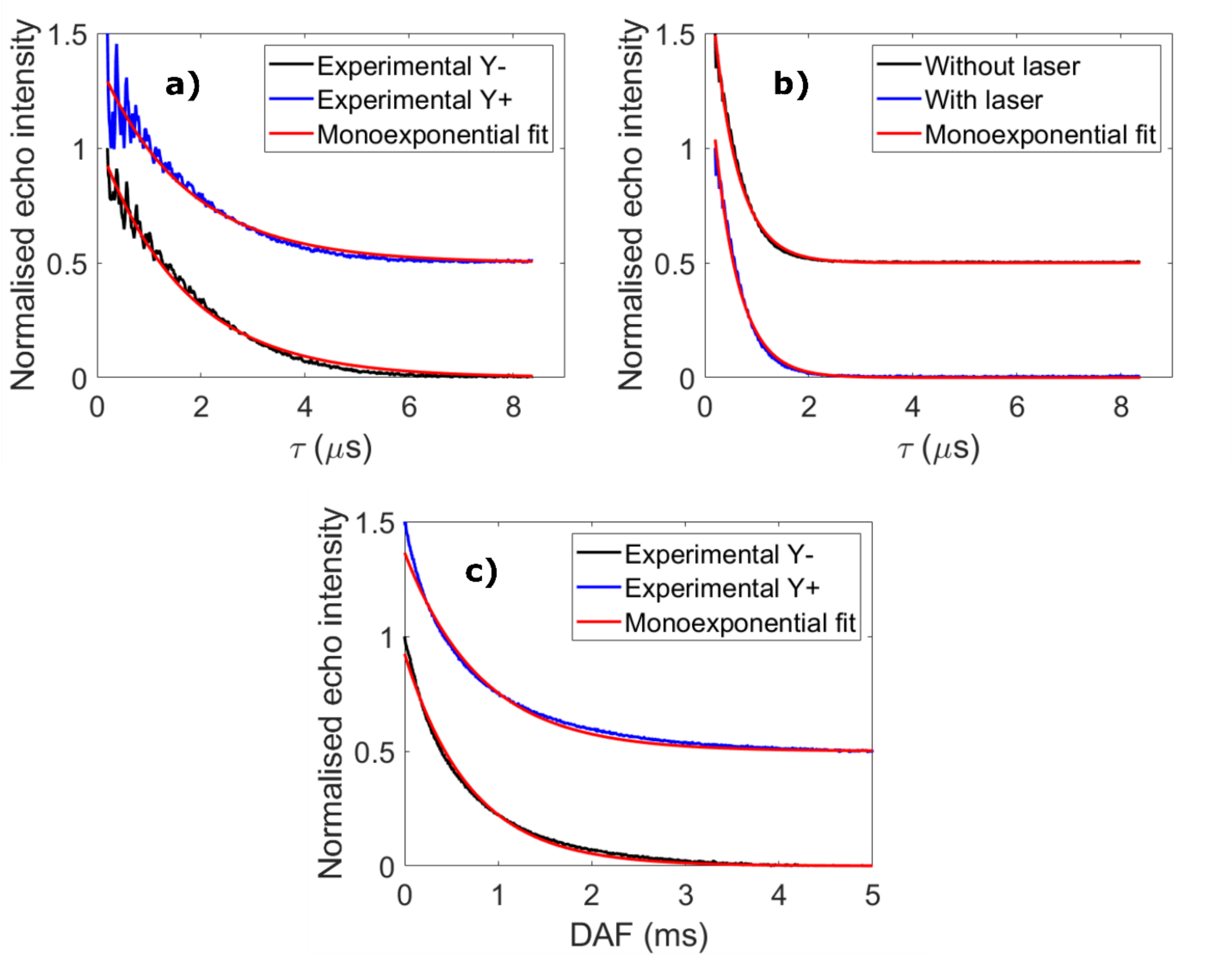

Figure S4. Relaxation experiments with molecule **[1]**. (a) Transverse relaxation experiments (black and blue) and corresponding monoexponential fits (red) measured on Y^- and Y^+ with T_2 values of (1.65 ± 0.03) μs and (1.68 ± 0.04) μs, respectively. (b) Transverse relaxation experiments (black and blue) and corresponding monoexponential fits (red) measured on the nitroxide maximum without and with laser excitation, with T_2 values of (0.48 ± 0.05) μs and (0.48 ± 0.04) μs, respectively. Delay after flash (DAF) experiment (black and blue) and corresponding monoexponential fits (red) measured on Y^- and Y^+ with lifetimes of (0.70 ± 0.005) ms and (0.82 ± 0.003) ms, respectively.

S3.2. DFT results

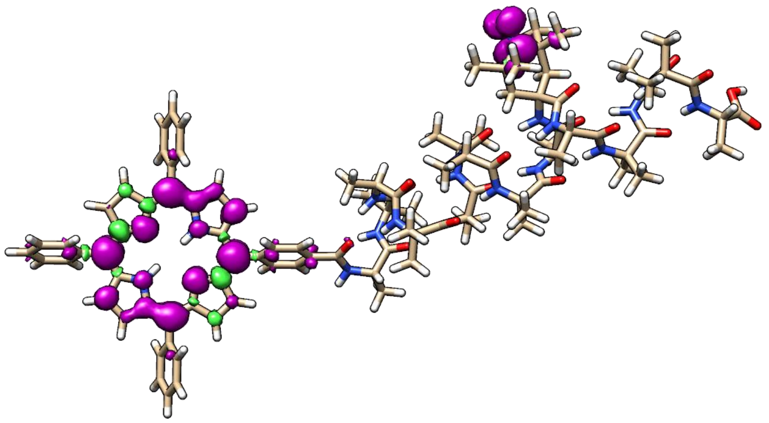

Figure S5. Electronic spin density in the DFT-optimized **[1]**, where the TPP moiety has been photoexcited to the first triplet state, calculated using Gaussian® 09 (B3LYP, EPR-II).

S3.3. Orientation-selective ReLaserIMD

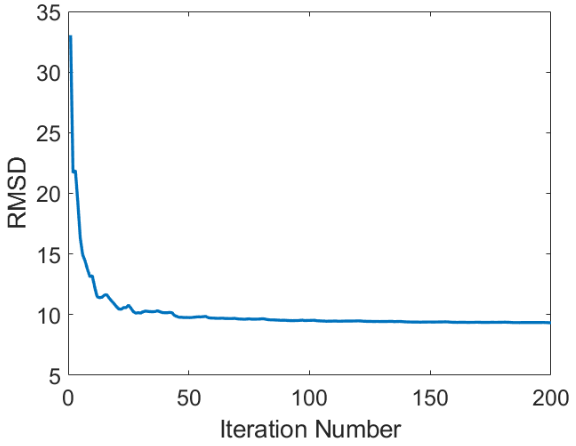

Figure S6. Root-mean-square deviation (RMSD) plot for the iterative process leading to the fits shown in Figure 2 (b).

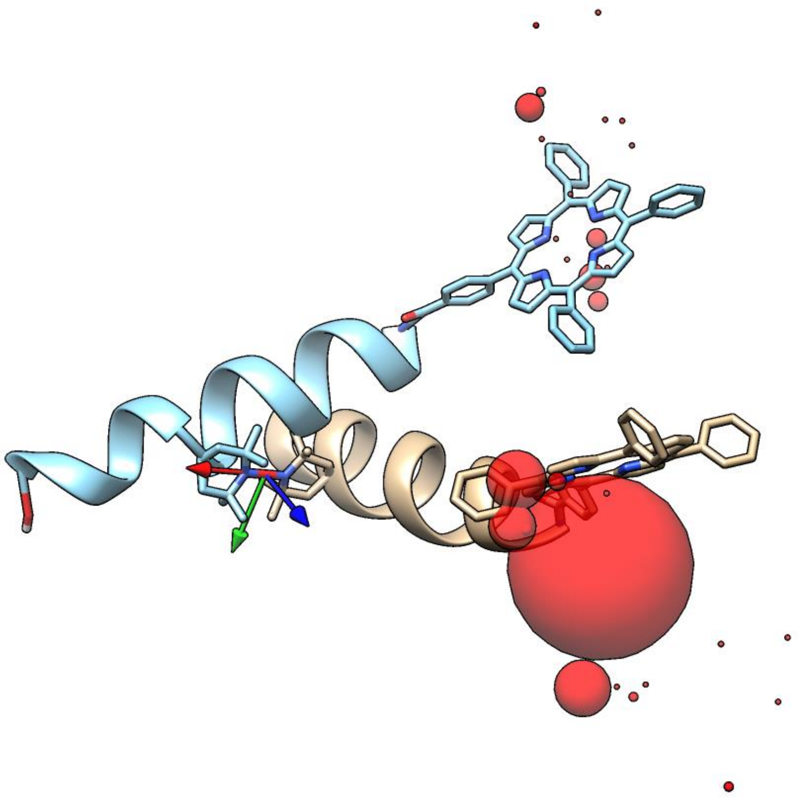

Figure S7. Different conformers of **[1]**. The taupe structure was determined by DFT optimization (Gaussian 09, PBE1PBE, 6-31g(d)). The blue structure has the TOAC in a secondary chair conformation, as predicted by.[14] The red spheres represent the different positions of the porphyrin center determined by the fits shown in Figure 2 (b), relative to the nitroxide g tensor frame (arrows, red = g_x, green = g_y, blue = g_z). The diameter of the sphere is proportional to the number of times a single porphyrin position contributes to the complete fit shown in Figure 2 (b).

S3.4. Frequency-correlated ReLaserIMD

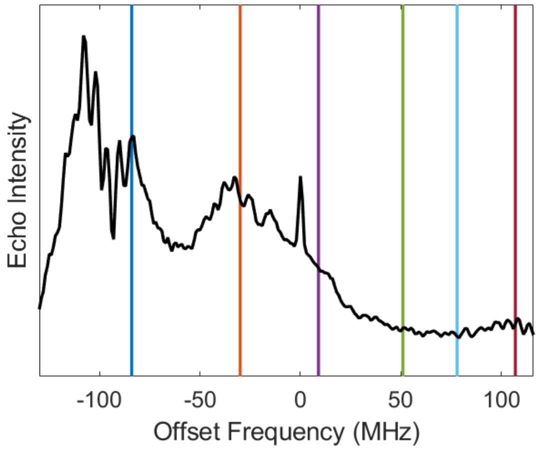

Figure S8. Fourier transform of the electron spin echo field swept spectrum of [1] after photoexcitation at 512 nm, also shown in Figure 3 (a). The experimental frequency values used for the FC ReLaserIMD experiment are indicated as vertical lines.

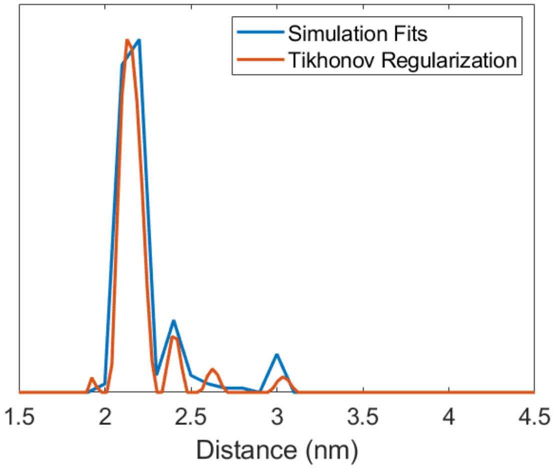

Figure S9. Interspin distance distributions from the simulation fits of the FC ReLaserIMD frequency slices shown in Figure 3 (c) and from Tikhonov regularization of the summed trace shown in Figure 3 (e).

S3.5. Orientation-selective LiDEER

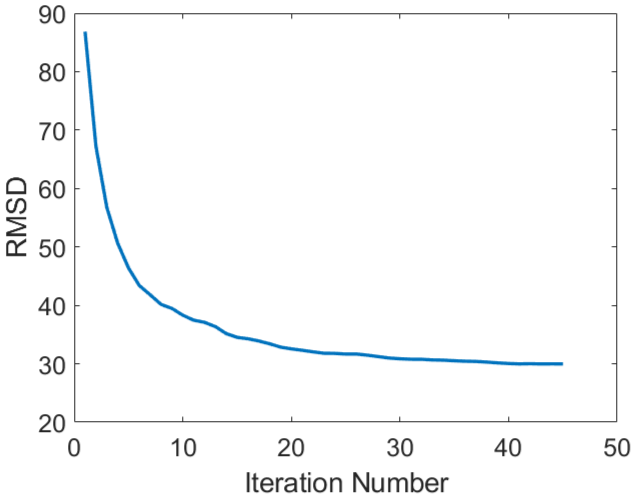

Figure S10. Root-mean-square deviation (RMSD) plot for the iterative process leading to the fits shown in Figure 4 (d-f).

REFERENCES

(1) Di Valentin, M.; Albertini, M.; Zurlo, E.; Gobbo, M.; Carbonera, D. Porphyrin Triplet State as a Potential Spin Label for Nanometer Distance Measurements by Peldor Spectroscopy. *J. Am. Chem. Soc.* **2014**, *136* (18), 6582–6585. https://doi.org/10.1021/ja502615n.

(2) Spindler, P. E.; Schöps, P.; Kallies, W.; Glaser, S. J.; Prisner, T. F. Perspectives of Shaped Pulses for EPR Spectroscopy. *J. Magn. Reson.* **2017**, *280*, 30–45. https://doi.org/10.1016/j.jmr.2017.02.023.

(3) Bieber, A.; Bücker, D.; Drescher, M. Light-Induced Dipolar Spectroscopy – A Quantitative Comparison between LiDEER and LaserIMD. *J. Magn. Reson.* **2018**, *296*, 29–35. https://doi.org/10.1016/j.jmr.2018.08.006.

(4) Stoll, S.; Schweiger, A. EasySpin, a Comprehensive Software Package for Spectral Simulation and Analysis in EPR. *J. Magn. Reson.* **2006**, *178* (1), 42–55. https://doi.org/10.1016/j.jmr.2005.08.013.

(5) Di Valentin, M.; Albertini, M.; Dal Farra, M. G.; Zurlo, E.; Orian, L.; Polimeno, A.; Gobbo, M.; Carbonera, D. Light-Induced Porphyrin-Based Spectroscopic Ruler for Nanometer Distance Measurements. *Chem. - A Eur. J.* **2016**, *22* (48), 17204–17214. https://doi.org/10.1002/chem.201603666.

(6) Monaco, V.; Formaggio, F.; Crisma, M.; Toniolo, C.; Hanson, P.; Millhauser, G.; George, C.; Deschamps, J. R.; Flippen-Anderson, J. L. Determining the Occurrence of a 310-Helix and an α-Helix in Two Different Segments of a Lipopeptaibol Antibiotic Using TOAC, a Nitroxide Spin-Labeled C(α)-Tetrasubstituted α-Aminoacid. *Bioorganic Med. Chem.* **1999**,

7 (1), 119–131. https://doi.org/10.1016/S0968-0896(98)00220-X.

(7) Pettersen, E. F.; Goddard, T. D.; Huang, C. C.; Couch, G. S.; Greenblatt, D. M.; Meng, E. C.; Ferrin, T. E. UCSF Chimera - A Visualization System for Exploratory Research and Analysis. *J. Comput. Chem.* **2004**, *25* (13), 1605–1612. https://doi.org/10.1002/jcc.20084.

(8) Frisch, M. J.; Trucks, G. W.; Schlegel, H. B.; Scuseria, G. E.; Robb, M. A.; Cheeseman, J. R.; Scalmani, G.; Barone, V.; Petersson, G. A.; Nakatsuji, H.; et al. Gaussian 09. Gaussian, Inc.: Wallingford CT 2009.

(9) Barone., V. Structure, Magnetic Properties and Reactivities of Open-Shell Species from Density Functional and Self-Consistent Hybrid Methods. In *Recent Advances in Density Functional Methods, Part I*; Chong, D. P., Ed.; World Scientific Publ. Co.: Singapore, 1996.

(10) Neese, F. The ORCA Program System. *Wiley Interdiscip. Rev. Comput. Mol. Sci.* **2012**, *2* (1), 73–78. https://doi.org/10.1002/wcms.81.

(11) Sinnecker, S.; Neese, F. Spin-Spin Contributions to the Zero-Field Splitting Tensor in Organic Triplets, Carbenes and Biradicals - A Density Functional and Ab Initio Study. *J. Phys. Chem. A* **2006**, *110* (44), 12267–12275. https://doi.org/10.1021/jp0643303.

(12) Lovett, J. E.; Bowen, A. M.; Timmel, C. R.; Jones, M. W.; Dilworth, J. R.; Caprotti, D.; Bell, S. G.; Wong, L. L.; Harmer, J. Structural Information from Orientationally Selective DEER Spectroscopy. *Phys. Chem. Chem. Phys.* **2009**, *11*, 6840–6848. https://doi.org/10.1039/b913085n.

(13) Marko, A.; Prisner, T. F. An Algorithm to Analyze PELDOR Data of Rigid Spin Label

Pairs. *Phys. Chem. Chem. Phys.* **2013**, *15* (2), 619–627. https://doi.org/10.1039/c2cp42942j.

(14) Elsäßer, C.; Monien, B.; Haehnel, W.; Bittl, R. Orientation of Spin Labels in de Novo Peptides. *Magn. Reson. Chem.* **2005**, *43* (SPEC. ISS.), 26–33. https://doi.org/10.1002/mrc.1692.
